# Autonomous field measurements of CO_2_ in the atmospheric column with the miniaturized laser heterodyne radiometer (Mini-LHR)

**DOI:** 10.1007/s00340-015-6172-3

**Published:** 2015-07-28

**Authors:** H. R. Melroy, E. L. Wilson, G. B. Clarke, L. E. Ott, J. Mao, A. K. Ramanathan, M. L. McLinden

**Affiliations:** American University, 4400 Massachusetts Avenue, Washington, DC 20016 USA; Laser Remote Sensing Laboratory, NASA Goddard Space Flight Center, 8800 Greenbelt Road, Greenbelt, MD 20771 USA; Global Modeling and Assimilation Office, NASA Goddard Space Flight Center, 8800 Greenbelt Road, Greenbelt, MD 20771 USA; Earth System Science Interdisciplinary Center, University of Maryland, College Park, MD 20740 USA; Microwave Instrument and Technology Branch, NASA Goddard Space Flight Center, 8800 Greenbelt Road, Greenbelt, MD 20771 USA

## Abstract

We present column CO_2_ measurements taken by the passive miniaturized laser heterodyne radiometer (Mini-LHR) at 1611.51 nm at the Mauna Loa Observatory in Hawaii. The Mini-LHR was operated autonomously, during the month of May 2013 at this site, working in tandem with an AERONET sun photometer that measures aerosol optical depth at 15-min intervals during daylight hours. Laser heterodyne radiometry has been used since the 1970s to measure atmospheric gases such as ozone, water vapor, methane, ammonia, chlorine monoxide, and nitrous oxide. This iteration of the technology utilizes distributed feedback lasers to produce a low-cost, small, portable sensor that has potential for global deployment. Applications of this instrument include supplementation of existing monitoring networks to provide denser global coverage, providing validation for larger satellite missions, and targeting regions of carbon flux uncertainty. Also presented here are preliminary retrieval analysis and the performance analysis that demonstrate that the Mini-LHR responds extremely well to changes in the atmospheric absorption.

## Introduction

Increasing atmospheric carbon dioxide (CO_2_) concentrations will exert a major influence on climate over the coming century. The rate of increase in atmospheric CO_2_ depends critically on both the magnitude of anthropogenic emissions and the ability of natural land and ocean carbon reservoirs to absorb this emitted carbon. Over recent decades, approximately 40 % of emitted anthropogenic CO_2_ has remained in the atmosphere, while the remainder has been stored in land and ocean sinks [1]. Despite advances in CO_2_ modeling and observing capabilities, the processes governing the flux of carbon between the atmosphere, land, and oceans are not well understood, leading to considerable uncertainty in projections of future climate change.

Surface CO_2_ measurements, made by a network of remote surface sites for several decades, have provided a strong constraint on the atmospheric CO_2_ growth rate. However, due to their remote locations, they provide limited information on the spatial distribution of carbon sources and sinks. Precise column CO_2_ measurements from satellites have been demonstrated to be useful for inferring fluxes [2–4], but the results can be strongly influenced by systematic errors contained in the data [5–7]. The ground-based TCCON has been critical in calibrating and validating satellite datasets from GOSAT and OCO-2 [2–4], and its independent utility for flux inference has also been demonstrated [8]. However, high instrument costs have limited the network size, and currently less than 20 sites exist globally.

The Mini-LHR demonstrated in this work could contribute to the global effort to reduce flux uncertainties by further validating satellite observations and supplementing measurements of ground-based in situ [5] and column networks [4]. Existing networks are characterized by notable gaps such as South America and Africa, which limits understanding of carbon fluxes in these regions. Existing satellites are also unable to see through clouds, which limits observations in persistently cloudy regions. While the Mini-LHR’s ability to observe is similarly limited by clouds, it can observe continuously throughout daylight hours, providing a greater opportunity to observe when breaks in clouds occur, or during times of the day when cloud obstruction is less frequent. The Mini-LHR has been designed to operate in tandem with AERONET (an established global network of nearly 500 ground-based instruments that measure aerosol optical depth [9]). This partnership provides a simplified path to global deployment into an established network. Tandem operation with AERONET may ultimately be beneficial for column CO_2_ measurements, as it has been shown that aerosols can modulate regional carbon cycles. Here we present field measurements of CO_2_ in the atmospheric column from the Mini-LHR operation at Mauna Loa Observatory (MLO), Hawaii.

## The Mini-LHR instrument design

**Fig. 1:**
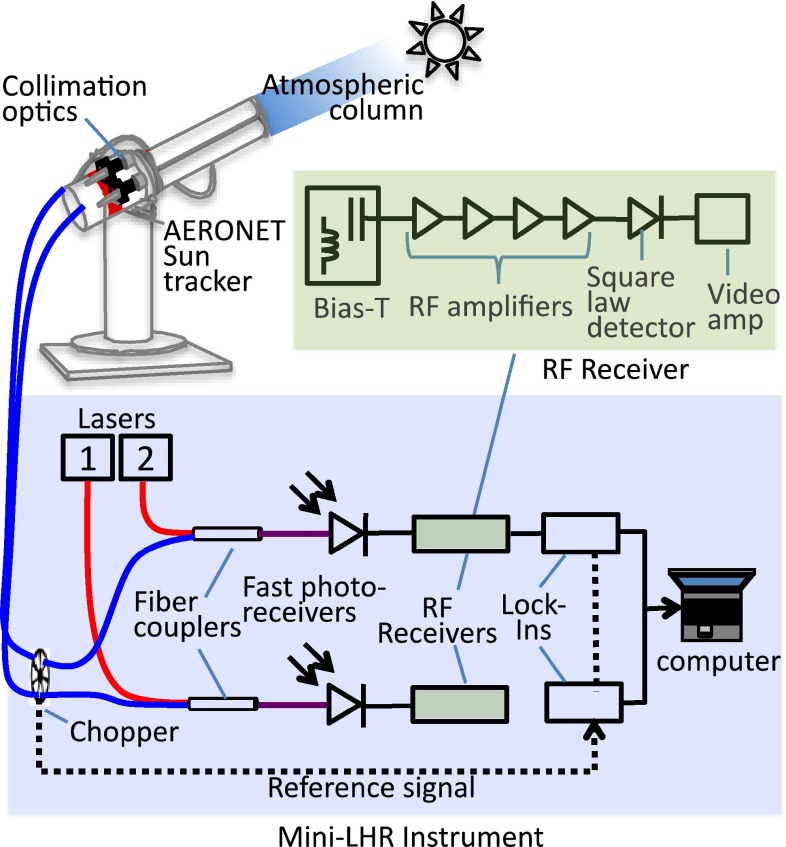
Mini-LHR instrument schematic (*shaded blue*) operated in tandem with an AERONET sun tracker (*upper left*). Sunlight that has passed through CO_2_ in the atmosphere is collected with collimation optics and then modulated with an optical chopper. The modulated sunlight is then superimposed with light from a DFB laser (laser 1) at 1611.5 nm in a single-mode fiber coupler, and mixed in a fast photoreceiver (InGaAs detector). A custom RF receiver (shaded green) detects and amplifies the resulting beat signal which is collected with a lock-in amplifier as the laser scans through the CO_2_ absorption feature. Components are also shown for a second channel that uses Laser 2 to measure an additional gas such as methane

The Mini-LHR is a simplified and compact version of a laser heterodyne radiometer [10–17] that measures shortwave infrared transmittance of trace gases in the atmospheric column by combining sunlight that has undergone absorption by the trace gas of interest with laser light at an adjacent wavelength to the absorption feature being measured. Mole fraction of the trace gas can then be retrieved from the collected spectra through a custom data-processing algorithm. The schematic in Fig. 1 shows a Mini-LHR arrangement for monitoring two separate gases simultaneously. This can be easily scaled to add more gas measurements with lasers selected to match the wavelength of the absorption feature (line) of interest. The CO_2_ line monitored here is centered at 1611.51 nm (6205.34 cm^−1^) and is the P(26e) transition of the (14°1)–(00°0) vibrational band centered at 1605.65 nm (6228 cm^−1^).

For each measurement, sunlight is collected with fiber-coupled collimation optics (Thorlabs F810SMA-1550 with ~0.2° field of view (FOV)). These optics are optimized to function in the near infrared (1050–1620 nm) and were chosen because they have a smaller FOV than the sun, which has a 0.5° FOV. The AERONET sun tracker (CIMEL Electronique model CE-318) has a pointing accuracy of ~0.1°. The collimator’s smaller FOV ensures that it does not drift off the sun throughout the day [18]. Zenith and azimuth stepping motors point the tracker to the calculated position of the sun (based on GPS coordinates and time of day), and then a four-quadrant detector updates the tracker’s position every 30 s [19]. Collected sunlight passes through a single-mode optical fiber (core diameter of 9.0 μm, 0.13 numerical aperture), is amplitude-modulated with an optical chopper (at 200 Hz), and then superimposed with light from an extended L-band-distributed feedback laser at 1611.51 nm (local oscillator) in a single-mode fiber coupler. Superimposed light is mixed in an InGaAs detector, producing an RF beat signal that is passed to the RF receiver.

Within the RF receiver, the beat signal passes through a bias-T (with a 50 ohm resistor) to separate the RF and DC outputs from the InGaAs detector. The RF output continues through a series of four amplifiers and is detected with a square-law detector to produce an output voltage that is then further amplified and low-pass-filtered with a video operational amplifier circuit (300-MHz bandwidth).

For each scan, the laser is scanned stepwise in frequency across the absorption feature, and the signal from the RF receiver is measured with a lock-in amplifier that is referenced to the optical chopper. Each scan takes approximately 1 min and consists of 55 points with a resolution of 0.002 nm over the feature and 0.01 nm across the wings, with an integration time of 500 ms for each point. Custom python software operated from a laptop computer controls the laser scanning and collects data from the lock-in amplifier.

1 $$E = \underbrace {{E_{L} e^{{i\omega_{L} }} }}_{{{\text{Laserlight}} + }} + \underbrace {{E_{S} (\omega )e^{i\omega t} }}_{{{\text{Sunlight}} + }}$$

2 $${\text{InGaAs{'} detector{'}}}\left\{ {\begin{array}{*{20}l} {I{ \propto }(E_{L} e^{{i\omega_{L} t}} + E_{S} (\omega )e^{i\omega t} ) \cdot (E_{L} e^{{ - i\omega_{L} t}} + E_{S} (\omega )e^{ - i\omega t} )} \hfill \\ { = \underbrace {{E_{L}^{2} + E_{S}^{2} }}_{{{\text{DC{'}}}}} + \underbrace {{2E_{L} E_{S} \cos [(\omega_{L} - \omega )t]}}_{{{\text{RF{'}}}}}} \hfill \\ \end{array} } \right.$$

3 $${\text{Square{'} law{'} detector{'}}}\left\{ {J(\omega ){ \propto }I_{\text{RF}}^{2} = 4E_{L}^{2} E_{S}^{2} (\omega )} \right.\cos^{2} [(\omega_{L} - \omega )t]$$

4 $${\text{Lock}}\;\% \;{\text{in}}\;({\text{amplifier)}}\left\{ {N = \int {J(\omega )} } \right.f(\omega - \omega_{L} ){\text{d}}\omega = 2E_{L}^{2} \int {E_{S}^{2} } (\omega )f(\omega - \omega_{L} ){\text{d}}\omega$$

**Fig. 2:**
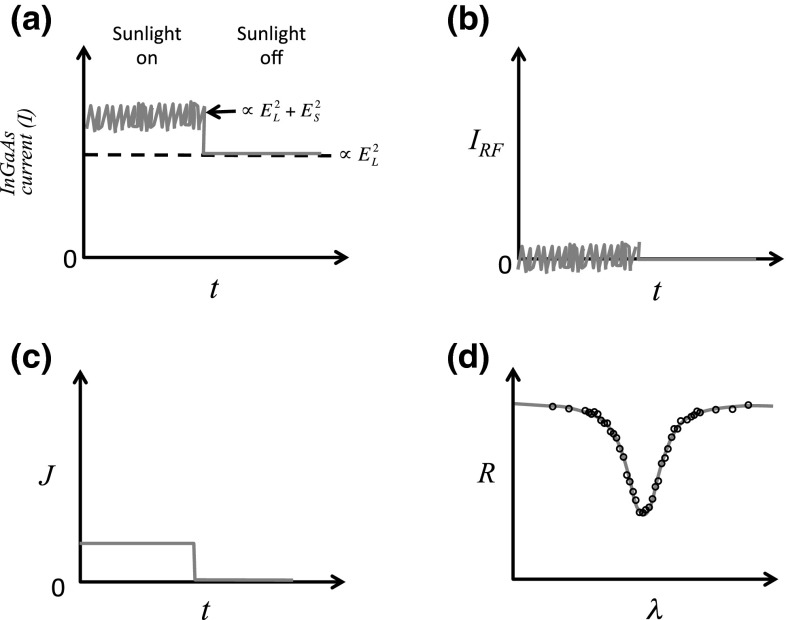
Pictorial descriptions of the heterodyne process. The interference of sunlight and laser light on the InGaAs detector is shown in (**a**). The same signal after the DC component has been removed is shown in (**b**) where *I*_RF_ is the RF component of the InGaAs detector current. The output of the RF receiver is depicted in (**c**) where *J* (which has a frequency-dependent response to sunlight) indicates the current output from the square-law detector as a function of time. The integrated signal, or irradiance *(R)*, is shown in (**d**) as the laser scans through the wavelengths of the absorption feature

The data collected from the lock-in amplifier can be traced back to the solar irradiances at different wavelengths. Here in Eqs. 1 through 4, we trace the formation of the beat signal from the interference of sunlight with laser light. Eq. 1 is the mixing of laser light and sunlight, where *E* is the combined electric field, *E*_*L*_ and *E*_*S*_ are the electric fields of laser and sunlight, respectively, *ω*_*L*_ is the laser frequency, *ω* is the optical frequency of the sunlight that beats with the laser light, and *t* is time as defined in an electric field. Eq. 2 is the measurement of the current *I*, by the fast photoreceiver which is proportional to the light intensity with both DC and RF components indicated. Eq. 3 shows the current output *J*, of the square-law detector which is proportional to the square of the RF component of the fast photoreceiver. The resulting measurement of the RF receiver output is shown in Eq. 4 as measured on the lock-in amplifier. The lock-in amplifier detects the demodulated signal referenced to the chopper frequency integrated over the instrument bandwidth. The output of the lock-in amplifier is proportional to the integrated solar irradiance within the instrument bandpass function *f*. An example of the implementation of this is shown with field data in Sect. 4. Figure 2 pictorially depicts the heterodyne process in these equations where Eq. 2 corresponds to (a) and its RF component to (b). Eq. 3 corresponds to (c) which is the output of the RF receiver, and Eq. 4 corresponds to (d) which is the result of an entire scan of CO_2_.

## Field site description

Data in this paper were taken at the MLO in Hawaii (Coordinates: 19.5362°N 155.5763°W, Elevation: 3397 masl) where Scripps Research Institute and NOAA have been monitoring in situ carbon dioxide since the 1950s [20, 21]. This field site provided a unique high-altitude platform to test the performance of the Mini-LHR, autonomous operation (the Mini-LHR operated unattended for the entire month of the field campaign, May 2013), component lifetimes, and modes of tandem operation with the AERONET sun tracker. The data were then used to develop the data analysis software to be discussed in Sect. 5.

**Fig. 3:**
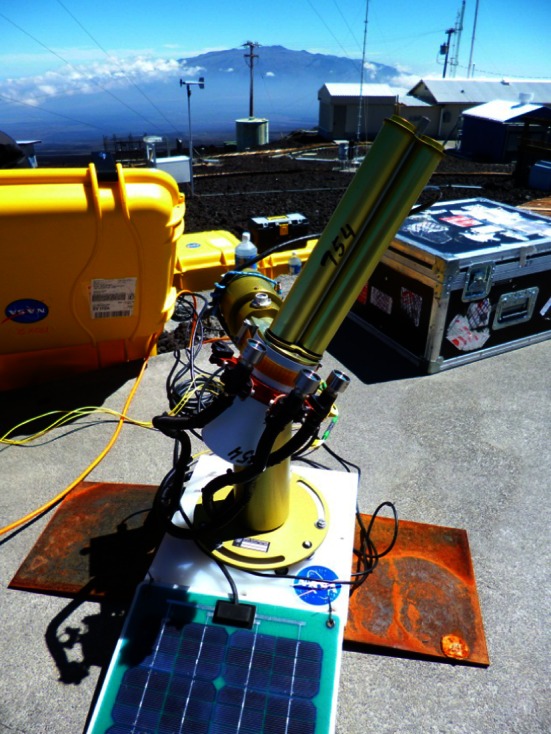
Mini-LHR instrument is shown at the Mauna Loa Observatory in Hawaii. MLO is the site of the longest running measurements of atmospheric CO_2_, begun in the late 1950s by Charles Keeling. MLO offers a unique testing environment for the Mini-LHR, as the altitude helps ensure the atmosphere sampled represents the “background” signal of the atmosphere

During the autonomous operation, the Mini-LHR was synchronized with the AERONET instrument operating in AUTO mode. In this configuration, data are collected within the ~2 min AERONET observation window which occurs approximately every 15 min during daylight hours. Figure 3 shows the Mini-LHR instrument operating in tandem with an AERONET sun tracker at MLO with Mauna Kea volcano visible in the background.

## Radiative transfer simulations

The measurement simulation implements a high-spectral-resolution radiative transfer model to calculate the transmittance through a given optical path in the atmosphere. The Line-By-Line Radiative Transfer Model (LBLRTM) [22, 23] was used with the HIgh-resolution TRANsmission molecular absorption (HITRAN) database [24] as input. Voigt line profiles were used to simulate the absorption lineshape, and line mixing [25] was added to the absorption calculations. Meteorological data, such as atmospheric vertical profiles of pressure, temperature, and water vapor, were taken from NASA’s Modern Era Retrospective Analysis for Research and Applications (MERRA) [26] and interpolated to measurement sites and time as ancillary data for absorption calculations. Data are drawn from the 6-h analyzed fields on the full model resolution and interpolated to measurement sites and time as ancillary data for atmospheric absorption calculations. The top of the atmosphere of the model data is around 76 km.

Both CO_2_ and H_2_O absorptions and Rayleigh extinction were included in the total absorption calculations and atmospheric refraction was also included, which can significantly change the light path length at large solar zenith angles.

**Fig. 4:**
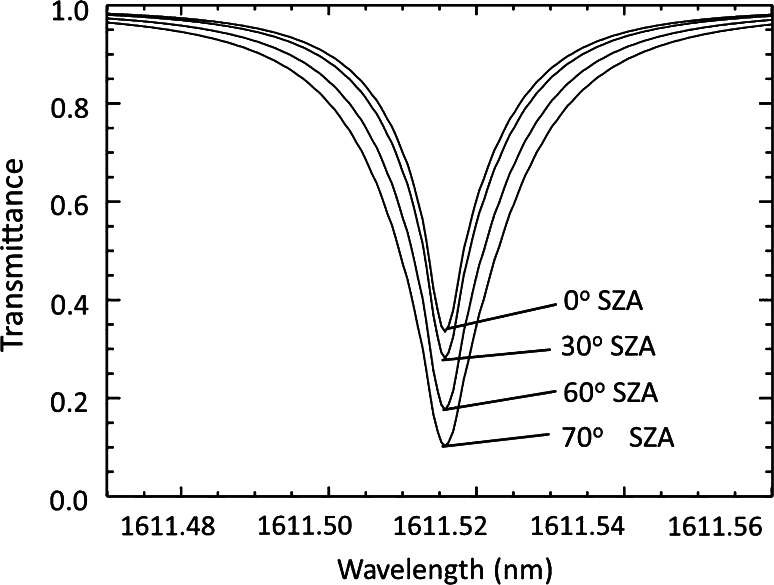
Simulated total column transmittance for the measurement line centered at 1561.515 nm at Mauna Loa, Hawaii, at 18 Z (UTC) on May 4, 2013. Each curve corresponds to the solar zenith angle of 0, 30°, 60° and 70°, respectively, and shows decrease in transmittance as solar zenith angle increases

**Fig. 5:**
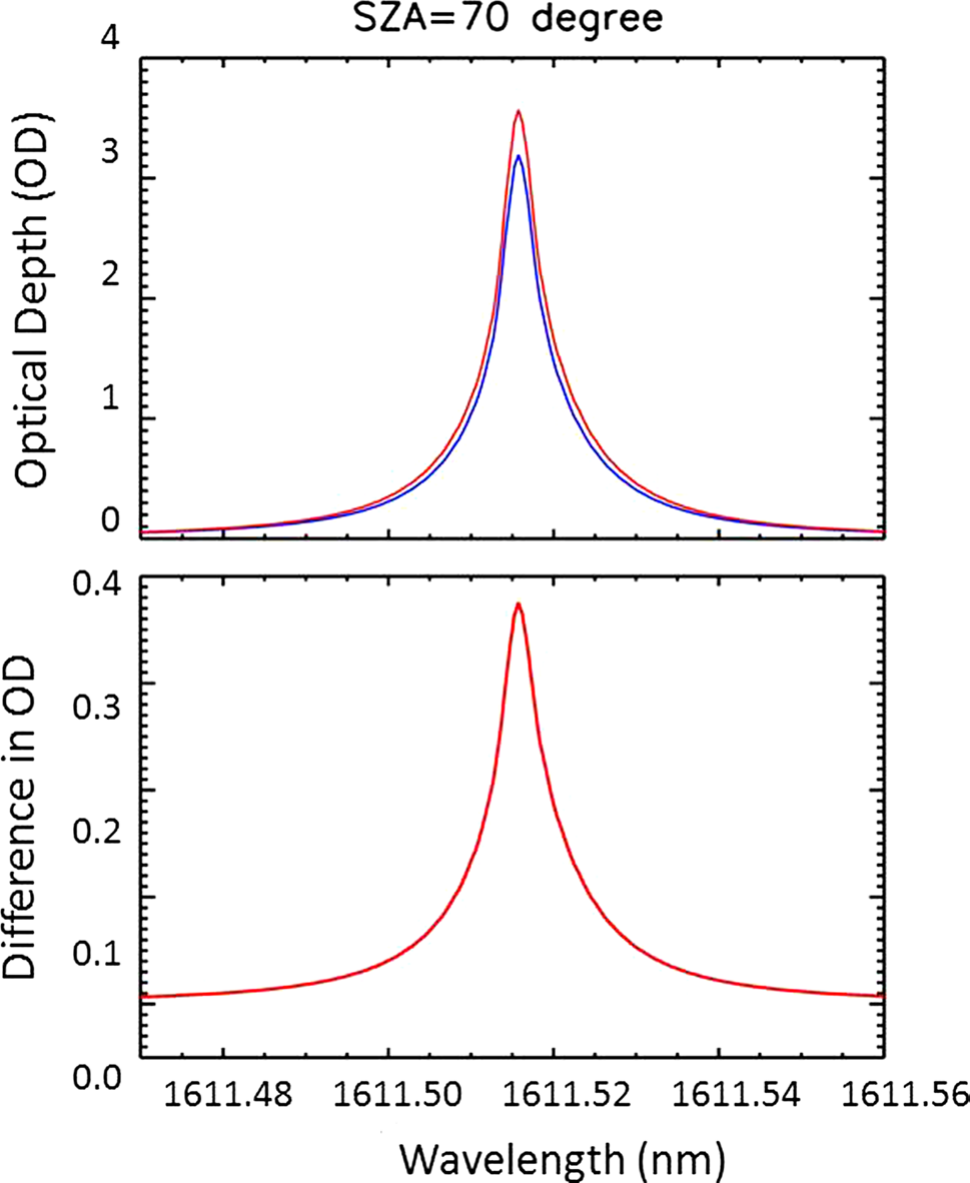
At large solar zenith angles, the impact of atmospheric refraction is more significant. The *top panel* shows the modeled total column optical depth for the same *line* plotted in Fig. 3 for an incident solar zenith angle of 70° at the top of the atmosphere, with atmospheric refraction (in *red*) and without (*blue*). The difference in these total column transmittances is shown in the *bottom panel* where a 10–15 % difference in OD is equivalent to 40–50 ppm

Figure 4 illustrates the total column transmittance as a function of incident solar zenith angle at the top of the atmosphere for the measurement line. Figure 5 shows the substantial effect of atmospheric refraction at a large solar zenith angle of 70°, which causes up to 0.35 in the optical depth at the Mauna Loa, Hawaii, or ranging 10–15 % cross the whole line. The effect should be greater if the column extends down to sea level.

## Data handling and analysis

Scans of CO_2_ collected by the Mini-LHR include date, time, laser wavelength, and lock-in signal. While data analysis is currently completed after the campaign, efforts are underway to incorporate data analysis into the acquisition software for real-time measurements.

There are four steps to retrieving the CO_2_ concentration. We first calculate the zenith transmittance for fixed atmospheric CO_2_ using a radiative transfer model. Then we scale the absorption depending on the solar zenith angle at the time of measurement. Next, we convolve the scaled transmittance with the instrument lineshape to factor in instrument line broadening. Finally, we fit this broadened lineshape to the LHR data and retrieve the CO_2_ concentration.

### Scaling the absorption for the appropriate solar zenith angle

The solar zenith angle was calculated based on the time of the day, the day of the year and the latitude and longitude [27]. The absorption was scaled by a factor of 1/cos(SZA), where SZA is the solar zenith angle. In addition, atmospheric refraction was also factored in [28], which mainly had an effect for SZAs above 60°.

### Convolution with instrument lineshape

Prior to fitting the calculated transmittance lineshape with the data, we need to factor the instrument response, *f(*Δ*ω)*, where Δ*ω* is the frequency difference between sunlight and laser light. The instrument response function of the Mini-LHR was characterized by mixing light from the scanning laser within the Mini-LHR instrument with light from a second, fixed laser (in lieu of sunlight) and recording the beat signal as a function of the scanning laser frequency as described in Clarke et al. [29]. In this characterization, the first laser was scanned from 1611.38 to 1611.64 nm, while the second laser was held constant at 1611.51 nm and the resulting instrument response bandwidth was 1.427 ± 0.005 GHz.

**Fig. 6:**
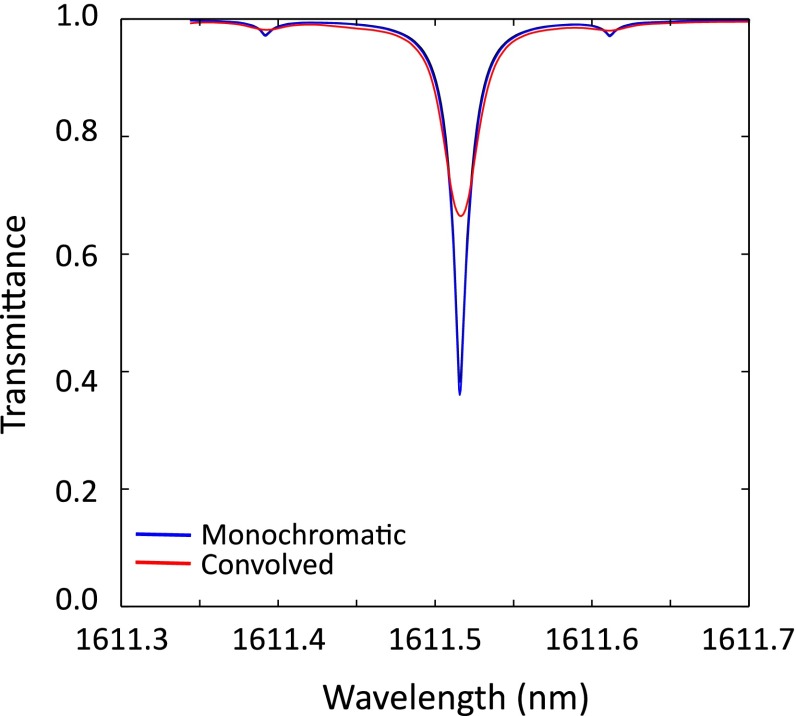
Effect of instrument line broadening: The 1.5-GHz heterodyne bandwidth causes the lineshape to be broadened. For this reason, prior to fitting data to calculations, we convolve the calculated transmitted lineshape with the instrument response as shown in this plot

For this work, we linearly interpolated the calibration data to a set of 1000 points evenly spaced at ~0.0005 nm (5 ppm–58 MHz). This interpolated calibration was then convolved with the radiative transfer modeled lineshape (which was generated at the same wavelength spacing), which broadened the lineshape. The convolution is illustrated in Fig. 6.

### Analysis of field data

The field data are read out of the lock-in amplifier in units proportional to the lock-in voltage signal. As shown earlier the final output is directly proportional to the sunlight within the instrument bandwidth of the reference laser frequency. To convert the field data to units of transmittance, we normalize the data by the mean signal of the off-line wavelengths (<1611.46 or >1611.57 nm). This measured transmittance is fitted to the calculations with two adjustable parameters—the wavelength offset and the CO_2_ concentration. The CO_2_ concentration parameter is a single scaling factor of the calculated transmittance, similar to Wunch et al. [30].

**Fig. 7:**
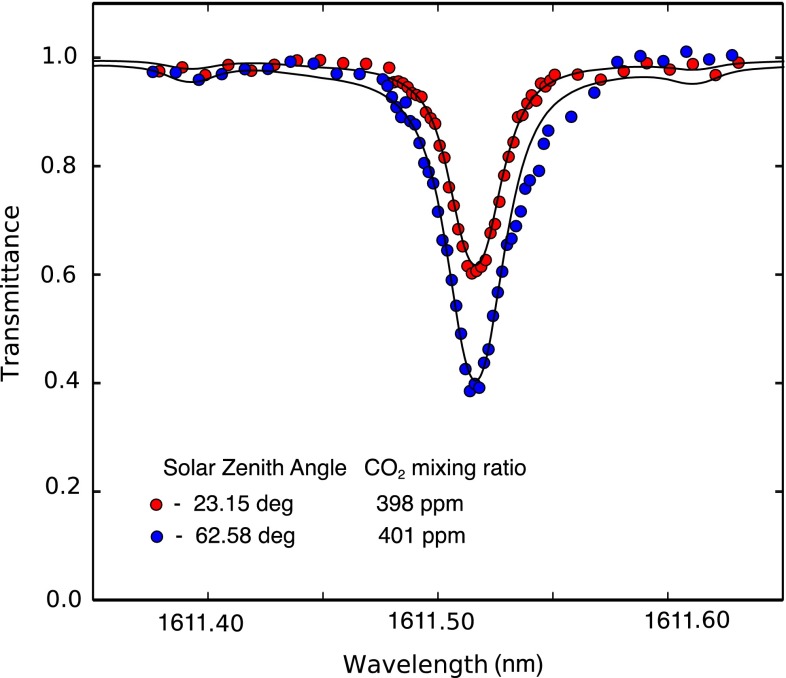
Shown are representative transmittance spectra of the CO_2_ absorption line centered at 1611.51 nm for single scans at two different solar zenith angles with the Mini-LHR at MLO. Experimental data (shown in *circles*) are fit with a simulated data (*lines*)

For the field campaign at MLO, we applied an overall calibration scale factor of 1.22 to the absorption. This factor was based on calibrating data to in situ MLO data on May 3, 2014. While the precise source of this scale factor is still being investigated, the factor did not vary during the duration of the field campaign. A sample fit of the calculated transmittance to a wavelength scan from the data is shown in Fig. 7.

**Fig. 8:**
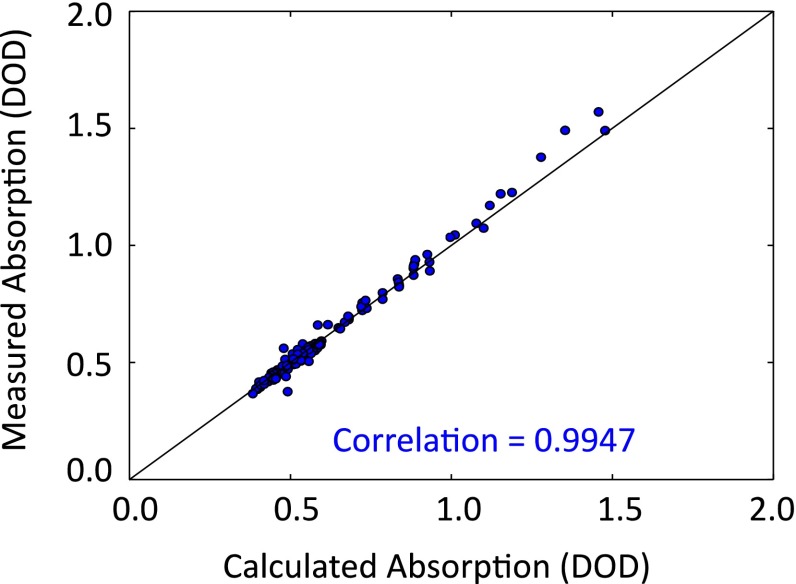
Baseline instrument performance—the absorption measured by the Mini-LHR is compared against calculations based on the solar zenith angle (for fixed CO_2_) for all cloud-free data taken at MLO in May 2013. The high correlation coefficient shows that the change in absorption due to change in optical path length is completely captured by the LHR. This is a prerequisite for CO_2_ concentration measurements. The absorptions are expressed in terms of the differential optical depth (DOD) between line center and off-line points

After fitting calculated lineshapes to individual wavelength scans, we then looked at a comparison of the calculated absorption based on atmospheric path length (assuming a uniform 400-ppm CO_2_ atmosphere) and the measured absorption (based on fit to data) for the entire campaign data May 6–25. This is plotted in Fig. 8 and shows an excellent correlation with *R*^2^ = 0.99. This shows that the Mini-LHR responds as expected to changes in the atmospheric absorption path length arising from the position of the sun.

## Discussion

Analysis of the field data has brought to light several necessary improvements to both the Mini-LHR instrument and the retrieval algorithm that will improve the sensitivity and reduce the instrument bias.

One improvement will be to implement a shorter scan time so that tens of scans can be made within the 2-min AERONET observation window. This will allow averaging of scans to reduce the random noise and improve measurement precision to the target of ~1 ppm. Data and analysis presented here has been of single, non-averaged scans which have a precision of ~6 % due to the signal-to-noise ratio and duration of the scan. Other passive techniques such as TCCON also average multiple scans to reach their target precision.

The results here have demonstrated the capability of the instrument which has been shown to respond extremely well to changes in the atmospheric path length and therefore absorption. It was found that refraction made a significant difference in the retrieval and should be included in pathlength calculation for better fitting—especially for high solar zenith angles. Also, the retrieval algorithm currently uses a simple uniform vertical CO_2_ profile. This could be improved by using a more sophisticated a priori vertical CO_2_ profile that varies with time of year and location, similar to the approach of TCCON’s retrieval [30]. Uncertainty in the atmospheric profiles (temperature, pressure, water vapor, and spectroscopic database) can contribute to errors in the retrievals.

## Conclusions

We have established the reliability of the Mini-LHR instrument using results from recent field testing at MLO in addition to an analysis framework developed to process data collected by the Mini-LHR. This is an essential first step to any new technology, technique, or instrument. The next step is to focus on reducing systematic errors and biases, and improving the retrieval algorithm, much the same way as TCCON and GOSAT have. For that we will be focusing on improving the scan time to achieve faster scanning, and editing the retrieval algorithm to include averaging. Additionally, we will include a commercialized low-noise RF receiver that is currently under development. These upcoming improvements will lower the instrument noise. Based on Fig. 6 of Clarke et al. [29], multi-scan averaging is expected to yield CO_2_ mixing ratio measurements of ~1 ppm if six or more scans are averaged. Future comparisons with TCCON and airborne in situ measurements will inform additional improvements to the instrument and retrieval algorithm.
